# The impact of cancer research: how publications influence UK cancer clinical guidelines

**DOI:** 10.1038/sj.bjc.6604405

**Published:** 2008-06-03

**Authors:** G Lewison, R Sullivan

**Affiliations:** 1 School of Library, Archive & Information Studies, University College London, Gower Street, London WC1E 6BT, UK; 2 Cancer Research UK, 61 Lincolns' Inn Fields, London WC2A 3PX, UK; 3 Department of Social Policy (Population, Health & Society), London School of Economics & Political Science, Houghton Street, London WC2A 2AE, UK; 4 European Cancer Research Managers Forum, London, UK

**Keywords:** guidelines, treatment, evidence, geography, funding

## Abstract

There has been a substantially increased interest in biomedical research impact assessment over the past 5 years. This can be studied by a number of methods, but its influence on clinical guidelines must rank as one of the most important. In cancer, there are 43 UK guidelines (and associated Health Technology Assessments) published (up to October 2006) across three series, each of which has an evidence base in the form of references, many of which are papers in peer-reviewed journals. These have all been identified and analysed to determine their geographical provenance and type of research, in comparison with overall oncology research published in the peak years of guideline references (1999–2001). The UK papers were cited nearly three times as frequently as would have been expected from their presence in world oncology research (6.5%). Within the United Kingdom, Edinburgh and Glasgow stood out for their unexpectedly high contributions to the guidelines' scientific base. The cited papers from the United Kingdom acknowledged much more explicit funding from all sectors than did the UK cancer research papers at the same research level.

It is increasingly being recognised that the quantitative evaluation of biomedical research cannot depend only on the counting of citations in the serial literature. They may measure academic influence, but the funders of such research are usually more concerned to see if it has had a practical benefit, especially to patients. One of the ways in which research can influence practice is through its contribution to the evidence base supporting clinical guidelines (Heffner, 1998; Gralla *et al*, 1999; Connis *et al*, 2000; Van Wersch and Eccles, 2001; Aldrich *et al*, 2003). These are increasingly being used across many countries in the routine clinical care of cancer patients. Most of them are published by national professional medical associations (e.g., Rizzo *et al*, 2002; Atwood *et al*, 2004; Makuuchi and Kokudo, 2006), but some are developed by governmental bodies (e.g., Pogach *et al*, 2004).

It is normal for such guidelines to have lists of references that comprise their evidence base. However, the quality of the evidence is sometimes doubtful (Ackman *et al*, 2000; Watine, 2002; Burgers and van Everdingen, 2004), and schemes have been devised to grade the quality of the clinical trials, which form a large part of the evidence base (e.g., Psaty *et al*, 2000; Liberati *et al*, 2001; Michaels and Booth, 2001; Hess, 2003; Guyatt *et al*, 2006). Even when the guidelines have been published, they are sometimes criticised as inadequate (Jacobson, 1998; Norheim, 1999; Walker, 2001), insufficient (Toman *et al*, 2001) or they may become outdated (Shekelle *et al*, 2001). There is also the question of whether the guidelines will actually be followed in clinical practice (Grol, 2001; Butzlaff *et al*, 2002; Bonetti *et al*, 2003; Bloom *et al*, 2004). The breadth of oncology practice (both patients and treatment modalities), the rapid evolution of new treatments and the often diverse interpretation of ‘evidence’ by health-care professionals mean many patients are treated with hospital-specific protocols rather than national guidelines. This situation is particularly acute in certain site-specific cancers, for example, lung (Sambrook and Girling, 2001).

A further cause of disagreement is the question of cost: a new drug may be clinically effective and better than existing drugs or a placebo, but so costly that an equivalent or greater health gain may be achievable by other means, for example, better screening to detect the disease at an early stage. This can cause considerable dissension and lead to lawsuits to make the drug available for particularly articulate patients (Dyer, 2006a), or from companies and patients' advocacy groups, which sometimes receive their subsidies (Dyer, 2006b). Lobbying of the UK National Institute for Health and Clinical Excellence (NICE) by pharmaceutical firms is now rife (Ferner and McDowell, 2006), and a US politician has adopted bully-boy tactics in his efforts to subvert evidence-based medicine (Kmietowicz, 2006). The cost basis of NICE's recommendations has also been criticised: the figure of £30 000 (€40 000, $60 000) per quality-adjusted life year appears not to have a scientific basis or to take account of the social costs of disease (Collier, 2008).

Despite all these criticisms, clinical guidelines are nevertheless gaining increasing recognition as the way forward. It does, therefore, seem worthwhile to treat them as an outcome indicator, even though a partial one, of the clinical impact of the research they cite. Several studies have analysed the evidence base of selected clinical guidelines (Grant, 1999; Grant *et al*, 2000; Lewison and Wilcox-Jay, 2003). They have established that the papers cited are very clinical (when positioned on a scale from clinical observation to basic research); that the UK guidelines overcite the UK research papers; and that the cited papers are quite recent, with a temporal distribution comparable to that of the papers cited on biomedical research papers. Research from other European countries seems to be cited about as much as would be expected on the UK clinical guidelines, but that from Japan and from most developing countries is almost totally ignored.

In this study, we examined three sets of the UK guidelines on a single subject, cancer, and the references on 43 different guidelines, almost all concerned with treatment rather than with prevention. The bibliographic details of the references were assembled in a file and compared with those of cancer research publications in the three peak years (1999–2001). The objective was to answer several policy-related questions:

- how do countries' relative presences among the cited references compare with their presences in cancer research?
- how many of the cited references are actually classifiable as cancer research?
- what is the research level (RL) distribution of these cited references compared with that of cancer research papers?
- are the cited references published in journals of high citation impact?
- how does the funding of the cited papers compare with that of cancer research overall?

The latter two questions need to take account of the finding that the references on clinical guidelines are much more clinical than other biomedical research.

## MATERIALS AND METHODS

### UK cancer guidelines and the analysis of their references

There are three sets of clinical guidelines commonly used in the United Kingdom:

- Published by the British Medical Association in *Clinical Evidence*. This takes the form of a book that is revised and extended every 6 months, but is also accessible on the Web (to people in the United Kingdom);
- Developed by the National Institute for Health and Clinical Excellence (NICE) for the National Health Service (NHS) in England and Wales, based on Health Technology Assessments (HTAs). Most of these last are available on the Web, but not all (although it is intended by NICE that they should be). They were used in the present study, because the references on the actual guidelines were usually not visible;
- Developed by the Scottish Intercollegiate Guidelines Network (SIGN) for use by the NHS in Scotland. All these are freely available on the Web

Only a minority of these guidelines and HTAs are applicable to cancer. The numbers are, respectively, 15, 18 and 10. Each of these 43 documents has a set of references, most of which are articles in peer-reviewed journals. A total of 3217 references were found and their details downloaded to file. Their addresses were parsed by means of a special macro so that the integer and fractional counts of each country were listed for each paper (a paper with two addresses in the United Kingdom and one in France would count unity for each on an integer count basis, but 0.67 for the United Kingdom and 0.33 for France using fractional counting). The RL of each paper was determined using the new system developed by Lewison and Paraje (2004), in which each journal is assigned an RL based on the presence of ‘clinical’ and ‘basic’ words in the titles of papers it has published on a scale from 1=clinical to 4=basic. In addition, the RL of groups of individual cited papers could be calculated with reference to their individual titles, and the presence of ‘clinical’ or ‘basic’ words within them. The potential citation impact (PCI) of each cited paper was also determined with reference to a file of Journal Expected Citation Rates provided by Thomson Scientific (London, UK). This gave the mean number of citations for papers published in a journal in a given year and cited in the year of publication and the 4 subsequent years.

Funding data for virtually all the UK papers (790 out of 796) were obtained from inspection of the acknowledgements to their funding sources in the British Library. Many of the papers had previously been looked up for the Research Outputs Database (Webster *et al*, 2003) or for other projects, and only 151 needed to be sought anew. The main comparator used to normalise the results of the analysis of the cited references was a file of world oncology research papers (Cambrosio *et al*, 2006). For the years 1999–2001, there were over 100 000 such papers, and their characteristics were used to see how the cited references compared with them, with due account being taken of the differences expected in mean RLs (the cited references being more clinical than oncology papers overall).

## RESULTS

### Time and research level distributions

**Figure 1:**
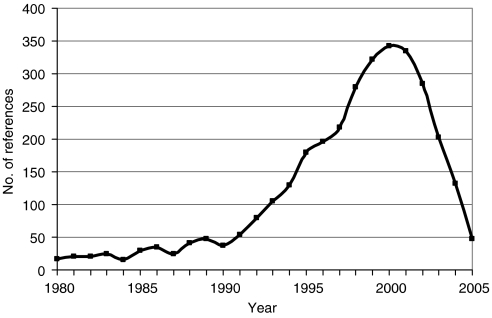
Time distribution of the 3217 references on the UK cancer clinical guidelines.

Figure 1 shows the distribution of the 3217 cited references by publication date. There is a clear peak in the year 2000, and 31% of all the references were published in the 3 years, 1999–2001, so this was the time period used for many of the comparisons with world oncology research.

**Figure 2:**
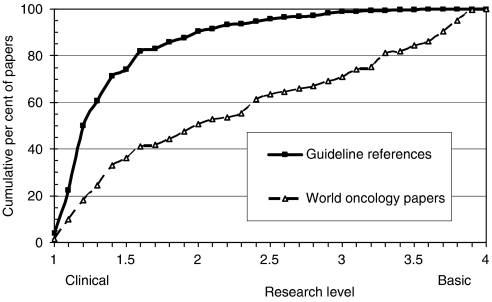
RL distributions (cumulative percentages) for references on cancer clinical guidelines (solid squares) and for oncology research in 2000 (open triangles).

Of the references classed as ‘articles’ or ‘reviews’, 88% were within the subfield of oncology as defined by Cancer Research UK (Cambrosio *et al*, 2006). This percentage remained sensibly constant over the period, 1994–2004. However, the references were in much more clinical journals than world oncology papers for the year 2000, the peak year for the numbers of references, see Figure 2. This result was obtained earlier (Grant *et al*, 2000; Lewison and Wilcox-Jay, 2003) but with a much simplified (and less accurate) method of categorisation of journals by RL. Of the 3217 papers, 2747 titles (86%) had either a ‘clinical’ or a ‘basic’ keyword, and the mean RL was 1.07, which is very close to the lower end of the scale (RL=1.0), and much below the mean RL based on all the papers in the individual journals (RL=1.43). This shows that the references were being published in journals that were relatively more basic than the papers themselves, and reinforces the message that the papers were, therefore, almost entirely clinical observation.

### Geographical analysis

**Figure 3:**
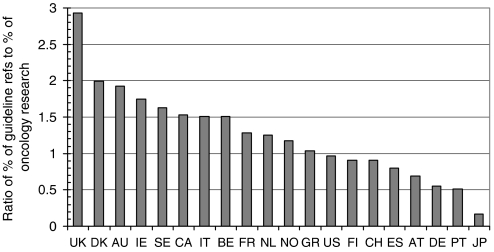
Ratio of countries' presence among the UK cancer clinical guideline references and their presence in world oncology research, 2000: fractional counts. Country codes as listed in Table 1.

The presence of 20 leading countries in oncology research for 2000 and in the references from the clinical guidelines is shown in Table 1, where the data have been shown on a fractional count basis. Figure 3 presents the ratio between a country's presence in the guideline references and its presence in oncology research, that is, the values shown in the last column of Table 1. As would be expected, the UK oncology research is cited more than expected from its presence in world oncology by a factor of almost 3, but several other European countries' work is also relatively overcited, notably that of Denmark, Ireland and Sweden. Although Italy, which is strong in clinical trials, shows to advantage, Germany is relatively much undercited compared with its presence in cancer research in recent years. Japanese work is almost ignored, but it is likely that the Science Citation Index, where most of the references were found, does not cover Japanese clinical journals. This, however, is only a small part of the reason for the paucity of Japanese references.

**Figure 4:**
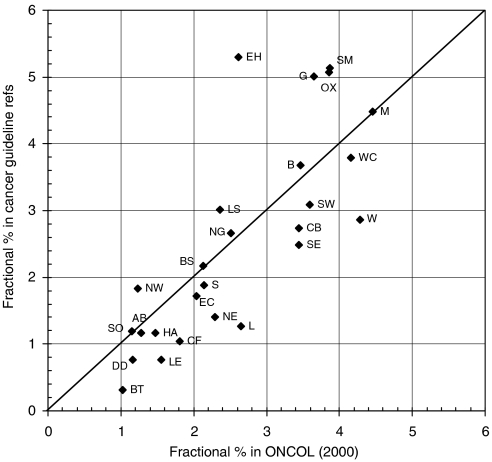
Scatter plot of the fractional count percentage presence of the leading 26 UK postcode areas within the UK papers cited on the UK cancer clinical guidelines plotted against their percentage presence in the UK oncology research outputs in 2000. Codes: AB=Aberdeen, B=Birmingham, BS=Bristol, BT=Belfast, CB=Cambridge, CF=Cardiff, DD=Dundee, EC=London EC (St Bart's), EH=Edinburgh, G=Glasgow, HA=Harrow, L=Liverpool, LE=Leicester, LS=Leeds, M=Manchester, NE=Newcastle upon Tyne, NG=Nottingham, NW=London NW (Royal Free), OX=Oxford, S=Sheffield, SE=London SE (Guys, Kings and St Thomas'), SM=Sutton and Cheam (Institute of Cancer Research), SO=Southampton, SW=London SW (St George's), W=London W (Imperial), WC=London WC (UCL).

Within the United Kingdom, certain cities showed relatively to advantage in terms of their percentage presence within the fractional UK total of 605 papers cited by the guidelines, compared with that in the 2332 UK oncology papers published in 2000. The analysis is conveniently carried out on the basis of postcode area, the first one or two letters of the UK postcode system, for example, B=Birmingham, CB=Cambridge. Figure 4 shows a scatter plot for the 26 leading areas (out of 124), accounting for about two-thirds of both totals. The spots above the diagonal line represent areas that are more frequently cited than expected, and vice versa. Among the former, EH=Edinburgh and G=Glasgow are prominent, in part because the SIGN guidelines overcite Scottish research papers, together with SM=Sutton and Cheam (the location of the Institute of Cancer Research) and OX=Oxford.

Table1 and Figure 3 show overall values, but an analysis can also be made of subsets of papers for groups of 2 or 3 years, chosen so that the four periods each have about 20% of the total cited references, see Table 2. For nearly all the countries, there are close similarities between the time trends, which suggest that the guidelines are rather consistent in the geography of their citing behaviour. Thus, Australia, Canada, Sweden, the United Kingdom and the United States have all shown a reducing presence in oncology research, and a reducing presence in the guideline references; Germany, on the other hand, has increased its presence in both (but is still much undercited). France and Japan increased their presence in both sets of papers, but it went down slightly during the latest period.

### Journal citation impact scores

The references cited tend to be published in high-impact journals. Table 3 shows that in each RL grouping, the guideline references are published in journals with a higher mean citation score (the PCI, of the papers) than world oncology papers from the year 2000.

The overall mean is higher, too, at 19.9 cites in 5 years compared with 13.4. The ‘superior performance’ of the guideline references occurs because a large number of them are published in the high-impact general journals, *The Lancet* (138 of them), *New England Journal of Medicine* (133), *British Medical Journal* (78) and *Journal of the American Medical Association* (50).

### The funding of the UK cited references

Of the 796 UK papers, all but 6 were found and inspected to determine their funding sources. These were taken both from the addresses (as for some organisations this is an indication of funding) and from the formal acknowledgements. For the purposes of this analysis, funding sources were grouped into five main sectors:

- UK government, both departments and agencies;
- UK private nonprofit, including collecting charities, endowed foundations, hospital trustees, mixed (academic) and other nonprofit. A subset of this sector is Cancer Research UK, and its two predecessors, the Cancer Research Campaign and the Imperial Cancer Research Fund;
- pharmaceutical industry, both domestic and foreign (it is often difficult to distinguish as some subsidiaries have considerable autonomy in the use of research funds), and including biotech companies;
- nonpharma industry;
- no funding acknowledged.

The remaining funding organisations are foreign governmental and private nonprofit sources, and international organisations, such as the European Commission (EC) and the World Health Organization (WHO).

The funding sources vary with the RL of the papers: the more clinical papers have fewer sources and the more basic papers have more. Table 4 shows the analysis for the UK papers in oncology in 1999–2001, and Table 5 shows the results for the UK papers cited on cancer clinical guidelines. For each RL group, an estimate has been made of the funding that would have been expected had they been typical of the UK cancer research, and in the last row there are given the ratios of observed-to-expected numbers of papers (integer counts) on the assumption that the cancer clinical guideline citations are typical of oncology, but with due allowance for the different RL distributions.

For example, the UK oncology papers in the first group (RL from 1.0 to 1.5) have the UK government funding on 11.1% of them, so it might be expected that there would be 0.111 × 544=60.4 government-funded papers among the corresponding group cited on cancer clinical guidelines. In fact, there were 149 such papers, showing that many more are government funded than might have been expected. When the totals for each of the six groups are added, it can be seen that the observed number of the UK government-funded papers is almost twice the predicted number. The observed total is still higher (× 2.5) for the pharma industry-funded papers, and a little lower for Cancer Research UK papers (× 1.8), for nonpharma industry papers (× 1.6) and the UK private nonprofit papers (× 1.3). Not surprisingly, there are many fewer ‘unfunded’ papers, the ratio of observed-to-expected numbers of papers being only just over half.

## DISCUSSION

The UK cancer clinical guidelines are sufficient in number and variety to provide a fair window on the impact of cancer research on clinical practice, not only in the United Kingdom, but in other leading countries, particularly in western Europe. We have seen that almost all the references (88%) are to papers that are within the subfield of cancer research. Because about one-third of the research supported by Cancer Research UK, in common with that of other medical research charities working in a particular disease area, is out with this subfield (most of this would comprise basic biology), it follows that little of this work can be expected to influence clinical guidelines – hardly a surprising conclusion, but nevertheless one that is worth stating.

Many of the guideline references are to papers in the US and the UK general medical journals – *The Journal of the American Medical Association*, *New England Journal Medical*, *British Medical Journal* and *The Lancet*. This is one reason, but by no means the only one, for the guideline references as a whole to be in high impact, and therefore well known, journals. It appears that if researchers want their work, particularly clinical trials, to be part of the evidence base for clinical guidelines, then it is desirable for them to publish in highly cited journals. Disproportionately, many of these papers will have been funded by government or the pharmaceutical industry, with charities also playing an enhanced role compared with cancer research overall. This highlights one pitfall of national guidelines in the context of research impact assessments; many important, high quality clinical trials – either because they are early phase or negative – will not make it into guidelines. The impact of research on national clinical guidelines is just one parameter that can describe the utility of health research (Kuruvilla *et al*, 2006).

When account is taken of the clinical nature of the work cited on guidelines, the big increase in the percentage of the papers that acknowledge funding – whether from government, charities or industry – is striking (Table 5). Many (37%) of these clinical papers with RLs greater than 1.5 are reports of clinical trials, and 85% of the latter acknowledge funding compared with 71% of the others. Cancer Research UK plays the biggest role, and supports over one-third of these trials, more even than the pharmaceutical industry as a whole, or the UK government.

The geographical analysis of the cited papers reveals that the UK papers have a threefold higher presence among them than in world cancer research. In part, this reflects the differences in cancer management between countries. Such overcitation also occurs on other scientific papers, so it is hardly surprising that it was found here. It might be expected that the UK guidelines, which aim to show which treatments are cost-effective, would reflect in particular the different financial basis of health-care provision in this country compared with that elsewhere, and so papers concerned with economics and costs would be even more overcited if they were from the United Kingdom. In fact, this does occur, but to a very minor extent (22% from the United Kingdom compared with 19% overall; the difference not being significant).

**Figure 5:**
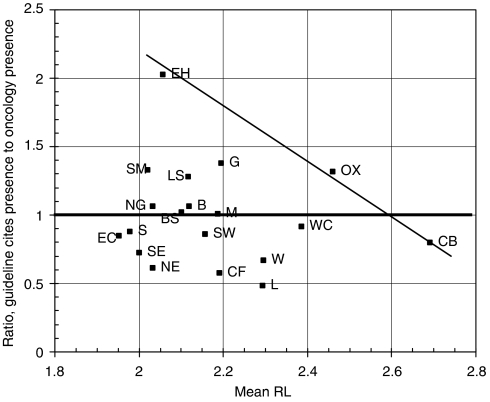
Comparison of the fractional count percentage presence of the 19 leading UK postcode areas with >50 cited papers cited by the UK cancer clinical guidelines divided by their presence in the UK oncology research in 2000 with the mean RL of their cited papers (scale: 1=clinical observation, 4=basic research). Area codes as listed in the legend to Figure 4.

The distribution of the cited papers within the United Kingdom differs from what might have been expected based purely on overall numbers and on the extent to which the cities carry out clinical observation rather than basic research. The simple comparison of Figure 4 needs also to take account of the mean RL of papers from each area, and, when this is done (Figure 5), a different pattern emerges, with EH=Edinburgh, OX=Oxford and CB=Cambridge forming an axis of excellence (on this indicator) and other areas' output being less cited on guidelines. The distance of the spots from this axis gives one indicator of the performance of the different centres, an imperfect one to be sure, as there will be other confounding factors not considered here, but nevertheless a useful complement to the traditional bibliometric criterion based purely on citation counts in the scientific literature.

There are in the database enough cited papers from a few other countries to enable a similar evaluation to be carried out for them. However, these data are inevitably skewed by being viewed through the prism of the UK clinical recommendations. It would be highly desirable to complement them with the results of similar exercises carried out in other countries with extensive sets of clinical guidelines, or at a European or international level. Then, provided the data were collected in exactly the same way, they could be pooled and a more international perspective on the utility of cancer research would emerge that research evaluators could employ. Such an activity could appropriately be coordinated by the European Cancer Managers' Research Forum, with all data contributors having also the right to gain access to the data provided by workers in other countries.

## Figures and Tables

**Table 1:** The fractional count outputs of 20 countries in oncology research in 2000 and in the references on the 43 UK cancer clinical guidelines and HTAs, their percentage presences and the ratio of the two percentages

**Country**	**ISO**	**Oncology research**	**Guideline references**	**Oncology references, %**	**Guideline references, %**	**Ratio**
Australia	AU	552	94	1.5	3.0	1.93
Austria	AT	402	25	1.1	0.8	0.69
Belgium	BE	353	47	1.0	1.5	1.51
Canada	CA	1056	143	2.9	4.5	1.53
Switzerland	CH	410	33	1.1	1.0	0.90
Germany	DE	2736	133	7.6	4.2	0.55
Denmark	DK	256	45	0.7	1.4	1.99
Spain	ES	646	46	1.8	1.4	0.80
Finland	FI	317	25	0.9	0.8	0.91
France	FR	1749	198	4.9	6.3	1.28
Greece	GR	270	25	0.8	0.8	1.04
Ireland	IE	70	11	0.2	0.3	1.75
Italy	IT	1939	259	5.4	8.2	1.51
Japan	JP	4601	67	12.8	2.1	0.16
Netherlands	NL	953	106	2.7	3.4	1.26
Norway	NO	188	20	0.5	0.6	1.17
Portugal	PT	42	2	0.1	0.1	0.51
Sweden	SE	627	90	1.7	2.8	1.63
United Kingdom	UK	2332	605	6.5	19.1	2.93
United States	US	12428	1068	34.7	33.7	0.97

ISO digraphs are used to denote the countries in Figure 3.

**Table 2:** Variation in time of the percentage presences of 10 leading countries in both the UK guideline references and the world oncology research; fractional counts

	**Guideline references**				**World oncology research**			
**Period**	**1995–1997**	**1998–1999**	**2000–2001**	**2002–2005**	**1995–1997**	**1998–1999**	**2000–2001**	**2002–2005**
AU	3.2	3.2	2.4	2.5	1.7	1.6	1.6	1.6
CA	5.3	4.7	4.5	4.0	3.0	3.0	2.9	2.8
DE	4.1	4.8	4.4	5.0	6.8	7.3	7.5	7.5
FR	5.9	7.4	6.6	6.0	5.1	5.2	4.7	4.6
IT	8.2	9.2	10.0	7.8	5.6	5.3	5.6	5.6
JP	1.7	2.6	2.7	2.5	11.2	12.6	12.4	11.6
NL	3.5	3.2	4.2	3.7	2.8	2.6	2.6	2.6
SE	3.4	2.3	3.1	3.1	2.1	1.8	1.8	1.6
UK	22.0	15.5	17.5	17.8	7.4	6.7	6.4	6.0
US	31.5	31.7	27.9	29.9	36.6	34.9	34.7	34.8

**Table 3:** Mean potential citation impact (PCI=expected cites in 5 year window) for world oncology papers for 2000 (oncology) and for guideline references

**RL**	***N* of oncology papers**	***N* of guideline references**	**PCI of oncology papers**	**PCI of guideline references**
*Clinical*				
1–1.5	12 465	2316	9.6	21.5
1.5–2	4958	511	10.2	14.3
2–2.5	4747	217	10.0	12.1
2.5–3	2941	114	14.6	23.5
3–3.5	4976	38	18.9	24.8
				
*Basic*				
3.5–4	5944	12	21.6	51.9

**Table 4:** Funding of the UK oncology research papers in 1999–2001, grouped by RL (integer counts); mean annual totals

**RL: ONCOL**	***N*(A)**	**% of A**	**GOV**	**GOV, %**	**PNP**	**PNP, %**	**CRUK**	**CRUK, %**
1–1.5	880	32	98	11	208	24	118	13
1.5–2	426	15	52	12	134	31	62	15
2–2.5	443	16	82	18	251	57	147	33
2.5–3	225	8	40	18	1247	55	55	24
3–3.5	330	12	77	23	189	57	99	30
3.5–4	452	16	163	36	300	66	163	36
Total	2756	100	511	19	1205	44	644	23
								
**RL: ONCOL**	***N*(A)**	**% of A**	**Pharm**	**Pharm, %**	**Ind'y**	**Ind'y, %**	**None**	**None, %**
1–1.5	880	32	53	6	25	3	527	60
1.5–2	426	15	39	9	17	4	200	47
2–2.5	443	16	71	16	20	4	96	22
2.5–3	225	8	22	10	70	3	48	21
3–3.5	330	12	43	13	17	5	43	13
3.5–4	452	16	65	15	20	4	37	8
Total	2756	100	294	11	106	4	950	35

A status=inspected papers; CRUK=Cancer Research UK; GOV=the UK government; Ind'y=other industry; Pharm=pharmaceutical industry; PNP=UK private nonprofit. Note: columns may not add correctly because of rounding.

**Table 5:** Funding of the UK papers cited by cancer clinical guidelines (G refs), grouped by RL (integer counts)

**RL: G refs**	**G refs**	**%**	**GOV-O**	**GOV-C**	**PNP-O**	**PNP-C**	**CRUK-O**	**CRUK-C**
1–1.5	544	69	149	60	198	129	142	73
1.5–2	127	16	26	16	49	40	39	19
2–2.5	83	11	13	15	46	47	33	28
2.5–3	19	2	4	3	13	11	9	5
3–3.5	13	2	2	3	5	7	3	4
3.5–4	1	0	1	0	1	1	0	0
Total	787	100	195	98	312	234	226	128
Obs/Calc			1.99		1.33		1.76	
								
**RL: G refs**	**G refs**	**%**	**Pharm-O**	**Pharm-C**	**Indy-O**	**Indy-C**	**None-O**	**None-C**
1–1.5	544	69	116	33	25	16	156	326
1.5–2	127	16	19	12	8	5	40	60
2–2.5	83	11	18	13	8	4	21	18
2.5–3	19	2	0	2	1	1	4	4
3–3.5	13	2	3	2	0	1	3	2
3.5–4	1	0	0	0	0	0	0	0
Total	787	100	156	62	42	26	224	409
Obs/Calc			2.53		1.63		0.55	

C=calculated on basis of ONCOL papers; O=observed number of papers. Columns may not add correctly because of rounding. Other column headings as in Table 4.
